# Inflammatory Granulomatous Corneal Disease: Ocular Granuloma Annulare

**DOI:** 10.7759/cureus.62582

**Published:** 2024-06-18

**Authors:** Aafreen Bari, Vaibhav Namdev, Baiju R Virani, Rajesh Sinha, Tushar Agarwal

**Affiliations:** 1 Ophthalmology, All India Institute of Medical Sciences, New Delhi, IND

**Keywords:** multisystem inflammatory syndrome, celiac disease and autoimmunity, inflammatory granulomatous corneal disease, cornea, granuloma annulare

## Abstract

Granuloma annulare is a well-known skin disease characterised by small papules arranged in a ring around a lesion with a normal atrophic centre. It may have variable clinical presentations and associations. Herein, we describe its novel ocular association with inflammatory granulomatous corneal disease (IGCD). It was observed in a young patient diagnosed with granuloma annulare. His symptoms included blurring of vision associated with photophobia in both eyes. There was marked stromal oedema with corneal haze at variable depths and mild anterior chamber flare. It resolved completely with topical steroids. This case discusses a unique manifestation of multisystemic IGCD with granuloma annulare that may co-exist with skin lesions. To the best of our knowledge, this unique entity has not been described in the literature previously.

## Introduction

Granuloma annulare (GA) is a non-infectious cutaneous granulomatous skin condition characterised by annular plaque-like skin lesions affecting various body parts. GA is typically a self-limiting disease and predominantly affects the lateral and dorsal areas of the hands and feet [1]. Ocular involvement in GA is a rare association with only a few case reports describing signs, such as uveitis and periocular granulomatous lesions [2-4]. Notably, there is no known corneal manifestation of GA existing in the literature.

The described case is an ocular manifestation of granuloma annulare which is a distinct type of multisystemic involvement of its primary skin disease. The multiple corneal lesions with stromal oedema, skin lesions and history of chronic diarrhoea which was diagnosed as celiac disease suggest a generalized multisystemic inflammation attributed to the pathophysiology of GA. This case represents the first documented instance of corneal involvement in patients with granuloma annulare. Understanding the clinical condition may offer valuable insights into its pathogenesis and potential treatment approaches. Further research and investigations are required to elucidate the complexities of this uncommon ocular manifestation and its management.

## Case presentation

**Figure 1:**
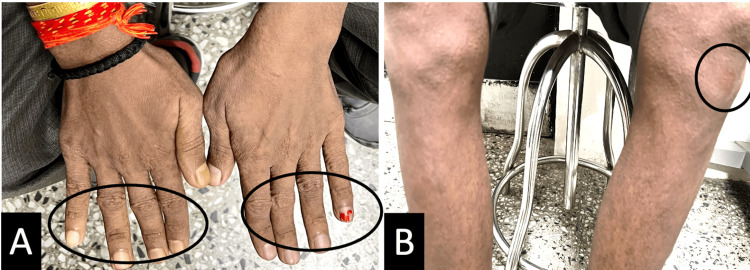
Composite image showing dermatological manifestations of granuloma annulare (A) Clinical photograph showing numerous papules on the dorsum of hands (marked). (B) Clinical photograph of legs with circinate rashes on the left leg (marked).

A 33-year-old man presented to the eye casualty department with complaints of blurred vision and photophobia in both eyes for two days. He had a few papules on the dorsum of the hands and around the knee for a month (Figure 1).

**Figure 2:**
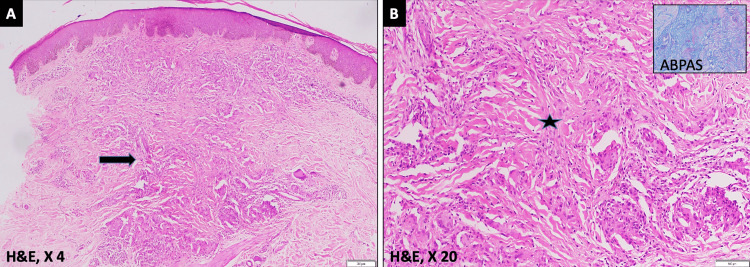
Skin biopsy of the case with histopathology image (A) Histopathology image shows mildly acanthotic epidermis with dermis showing a large granulomatous reaction (arrow) with central collagen degeneration and few dispersed multinucleate giant cells. (B) Higher magnification revealed central collagen degeneration (star) with interspersed histiocytes and giant cells. The inset image shows a special stain (Alcian blue periodic acid - Schiff) which indicates the presence of bluish mucin within the granuloma

He had recently been diagnosed with granuloma annulare from the dermatology department based on his clinical presentation and the diagnosis had been confirmed through a lesion biopsy (Figure 2).

**Figure 3:**
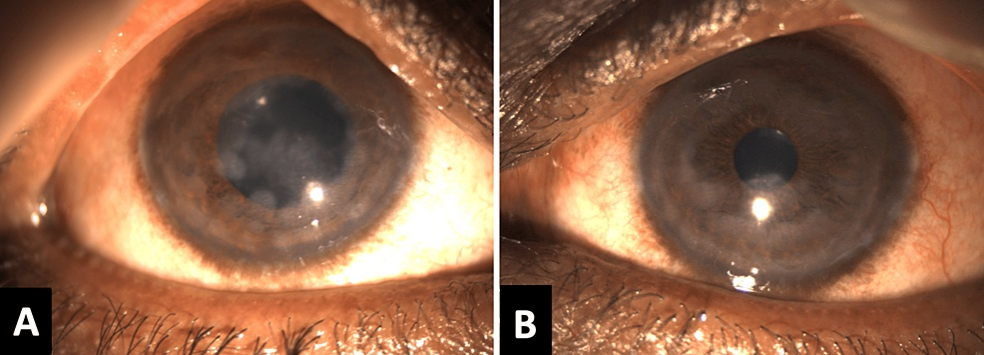
Slit-lamp photograph of both eyes on presentation (A) Right eye and (B) left eye showing numerous corneal opacities dispersed in a circular fashion sparing the visual axis.

There was no history of previous redness in the eyes, trauma, fever, itching or similar complaints in the family. On ocular examination, his visual acuity in both eyes was log MAR 0.30 and intra-ocular pressures were 10 mmHg and 12 mmHg in the right and left eyes, respectively. He had bilateral mild conjunctival congestion with numerous irregular-shaped nebular corneal opacities sparing the visual axis and mild stromal oedema (Figure 3).

There were no corneal epithelial defects and keratic precipitates. There was a mild anterior chamber (AC) reaction with 0.5+ AC cells and mild AC flare. Pupillary reactions were brisk. The lens was clear and the posterior segment examination was normal. On examination, his corneal sensations were diminished bilaterally. Anterior segment optical coherence tomography (AS-OCT) revealed discrete, multiple, hyper-reflective foci distributed at variable depths in the anterior and mid-stroma. The corneal thickness was increased bilaterally. The right and left eyes had corneal thicknesses of 602 and 620 micrometres, respectively.

The tear sample was negative for herpes simplex virus-1 (HSV-1) on polymerase chain reaction (PCR). The patient was advised to undergo triple viral marker testing (HIV, hepatitis B and hepatitis C) and chest X-ray, which were normal. There was no history of weight loss or any long-standing disease.

The patient was also referred to the gastroenterologist for chronic diarrhoea for which he was medically managed. To investigate the underlying cause, blood tests evaluating immunoglobulin levels along with duodenal biopsy were done. The serum Ig A levels were low. The duodenal biopsy showed a 1:1 C:V ratio at places with a high number of intra-epithelial lymphocytes. Crypt hyperplasia was present with lamina propria inflammation, moderate luminal parasite and absent impression. The features were suggestive of moderate villous abnormality with increased intraepithelial lymphocytes. On combining the clinical symptoms with the reports, a diagnosis of celiac disease was made.

The patient was prescribed topical steroids (prednisolone phosphate eye drops 1% four times a day, tapered over three weeks) for both eyes. For the skin lesions, he was advised oral steroids (betamethasone sodium phosphate tablets of 5 mg OD tapered over six weeks) and oral hydroxychloroquine (300 mg daily) along with antacids, calcium and cholecalciferol supplements. At the one-week follow-up, the patient's ocular symptoms improved markedly. His visual acuity improved to log MAR 0 and corneal thickness decreased bilaterally.

**Figure 4:**
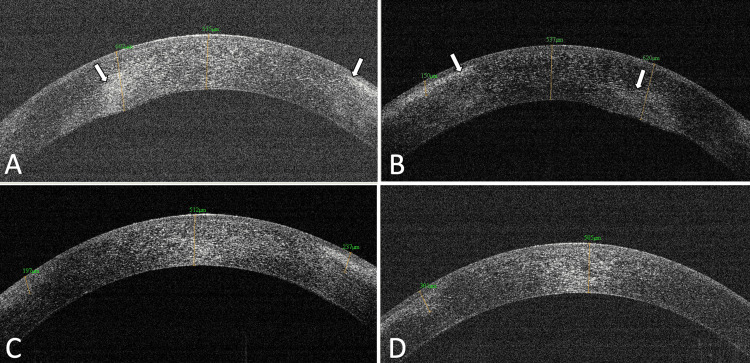
Anterior segment optical coherence tomography of both eyes showing improvement in corneal thickness from presentation to the subsequent one-month follow-up Anterior segment optical coherence tomography at the presentation of (A) the right eye with a maximum corneal thickness of 602 microns and (B) the left eye with a maximum corneal thickness of 620 microns with stromal oedema and corneal haze at various levels (arrows). At one month, (C) the right eye with a corneal thickness of 512 microns and (D) the left eye with a corneal thickness of 505 microns showed compact corneal stroma with corneal opacities at variable depth.

His skin problems also improved gradually, with the use of oral steroids. The patient attended follow-ups for a year and there were no dermatologic, ocular or systemic recurrences (Figure 4).

## Discussion

Granuloma annulare is a benign, non-infectious, cutaneous granulomatous condition, typically displaying a self-limited course. It is distinguished by annular circinate plaque-like skin lesions, predominantly distributed on the lateral and dorsal aspects of the hands and feet. Granuloma annulare encompasses various clinical variants, including the localised, generalised, subcutaneous, patch, and perforating forms. A few systemic disorders like type I and II diabetes mellitus and hypothyroidism along with various viruses like HSV, adenoviruses, HIV, and even SARS-Cov-2 have been described to be associated with granuloma annulare. Though the pathogenesis is not very clear, it has been described that they may act as a triggering factor for the condition at the immunological level [5,6].

Histologically, a granuloma is made of degenerated collagen, mucin and lymphocytes. The term granuloma annulare is based on similar findings observed in the histopathology of skin lesions of these patients. Among ocular manifestations, corneal signs have not been described previously in a patient with granuloma annulare. In this report, corneal findings have been termed as inflammatory granulomatous corneal disease assuming them to have common etiopathogenesis as the lesions of the skin. This is due to the concurrent occurrence of both skin and corneal involvement at presentation. A corneal biopsy would have established a definite pathological diagnosis, however, it was not done owing to good visual acuity at presentation and evident clinical improvement with topical steroids in the patient. It is one of the lacunae of the report.

The differential diagnosis in this case, presenting with inflammatory corneal granulomatous disease along with concurrent skin lesions and diarrhoea, includes multi-systemic auto-immune diseases that may be due to immunological reasons or triggered by a viral infection. The diagnosis is mostly clinical in cases of viral keratitis and keratouveitis. The described case presented with numerous areas of corneal haze with geographic distribution and variable depths of corneal stromal involvement. The clinical presentation was unlikely of adenoviral keratitis which classically presents as coin-shaped sub-epithelial infiltrates. Herpes simplex keratitis was a close differential diagnosis. However, it usually presents unilaterally. The tear PCR for HSV was sent and it showed negative results. Despite the negative tear PCR test, HSV keratitis could not be ruled out completely owing to the low sensitivity of the test [7]. This is another lacuna of the report.

Ocular involvement in GA is an exceedingly rare phenomenon, with only a few case reports highlighting ocular manifestations limited to uveitis and periocular granulomatous lesions. Notably, there is an absence of documented corneal involvement in GA within the existing literature. The described case is unique in describing the corneal manifestations of the multisystemic disease considering interplay at the immune level and involvement of multiple organs. The treatment involved the use of targeted and systemic steroids to subside the underlying inflammation. Encouragingly, the case exhibited a favourable response and did not relapse, resulting in symptomatic improvement and effective disease control. An integrated approach by a dermatologist, an ophthalmologist and a physician helped to effectively treat all systemic complaints of the patient. However, more cases need to be studied to understand the pathophysiology and appearance of clinical signs at a deeper level.

## Conclusions

Granuloma annulare may present with multisystemic inflammatory disease involving the skin, gastrointestinal tract and the eye. The inflammatory granulomatous corneal disease may present with blurred vision, photophobia and corneal stromal haze involving anterior and mid-stroma. Topical steroids are the main line of treatment for patients of inflammatory granulomatous corneal disease The visual prognosis is generally good with low recurrence rates. Comprehensive multisystemic evaluation and treatment are vital to treat all aspects of the disease spectrum.
